# Eating Disorder Symptoms and Alcohol Use Among Adolescents in Substance Abuse Treatment

**DOI:** 10.4137/sart.s3354

**Published:** 2009-11-13

**Authors:** Janelle E. Arias, Josephine M. Hawke, Albert J. Arias, Yifrah Kaminer

**Affiliations:** 1 Department of Child and Adolescent Psychiatry, Yale Child Study Center, New Haven, CT 06510, USA.; 2 Alcohol Research Center, Department of Psychiatry, University of Connecticut Health Center, Farmington, CT 06030, USA. Email: hawke@psychiatry.uchc.edu

**Keywords:** alcohol use disorders, substance use, eating disorders, adolescents, treatment, comorbidity

## Abstract

**Objective:**

To examine the relationship of eating disorder (ED) symptoms with the severity of alcohol use among adolescents in treatment for alcohol and other substance use disorders (AOSUDs).

**Method:**

A sample consisted of 177 adolescents who participated in outpatient AOSUD treatment programs in Connecticut. Chi square tests, one-way ANOVAs and Pearson’s correlation coefficients were used to describe the prevalence and correlates of any eating disorders, and the related symptoms. Multivariate regression was used to test the associations between ED symptoms and alcohol consumption.

**Results:**

26.4% of the participants had at least one ED symptom, with the highest number of symptoms occurring in females. The number of ED symptoms was associated with increases in the number of times that they became intoxicated in the year before entering treatment, the number of alcohol-related social problems, and the number of alcohol-related physical symptoms after taking into consideration the effects of age and gender.

**Conclusions:**

The prevalence of symptoms of EDs is high in adolescents with AOSUDs, with the number of ED symptoms correlating with increased alcohol consumption. Further studies on the course and treatment of adolescents with AOSUDs and symptoms of EDs are warranted.

## Introduction

Alcohol and other substance use disorders (AOSUDs) and eating disorders (EDs) in adolescence are public health concerns with personal and financial costs for adolescents, families, and their communities. Although high rates of psychiatric comorbidity have been well-documented among adolescents with AOSUDs,^1^–^6^ surprising little research has focused on EDs and related symptoms among substance-using adolescents.

Comoridity of the two disorders complicates clinical presentation and increases the risks of delinquency, suicidal behaviors, mortality, and family problems.^7^–^11^ The presence of ED symptoms has been seen to affect both the course of the development of AOSUDs,^9^,^12^–^15^ as well as their treatment.^16^–^18^ Particularly noteworthy, Franko and colleagues^12^ conducted a prospective study of adult women with EDs which found that a substantial number of women who initially present with EDs later develop alcohol use disorders.

Epidemiological studies indicate that eating disorders are fairly rare in the general population. Less than 1% of adolescents in the general population are estimated to have anorexia nervosa (AN) and less than 5% are estimated to have bulimia nervosa (BN).^19^–^21^ Rates of EDs among adolescents are typically higher among females than males and subthreshold presentations are most common.^22^ Binging behaviors are the most prevalent type of ED symptom observed among adolescents. In a study of 1,728 high school students, Killen and colleagues^23^ estimated that about 13% of the adolescent population exhibits purging behaviors, with typically higher binging behavior among females than males (16.2% vs. 5.2% respectively).

Prevalence rates of AOSUDs in adolescents with EDs and/or ED symptoms are much higher than in the general.^14^,^24^–^28^ Lewinshon and colleagues^29^ reported that 13% of a sample group of high school students who met criteria for subthreshold EDs had substance use disorders, compared to less than ten percent of a comparison group. In a study of adolescent girls, Stice and colleagues^30^ reported that 4.64% of the sample met broad diagnostic criteria for an eating disorder. Among those with EDs, 37.8% also met diagnostic criteria for a substance use disorder.

Similarly, rates of EDs are also higher among substance users.^24^,^25^,^27^,^28^ Increased rates of alcohol use have been consistently documented in both adolescents and adults with EDs.^28^,^31^,^32^ There is a significant association between the number of drinks reported at a sitting and the risk of having or developing an eating disorder.^33^ In one study examining female inpatients ages 13–38, Grilo and colleagues^34^,^35^ found higher rates of subthreshold EDs and ED Not Otherwise Specified (EDNOS) among inpatients with AOSUDs than among those without.

Several studies report associations between binge eating and bulimia and alcohol use. In a meta-analysis, Gadella and Piran^26^ found that 41 studies published between 1985 and 2006 reported consistently significant associations between disordered eating patterns and alcohol use disorders. Significant associations have been observed between AOSUDs and BN, purging, and binge eating, though not for AN, and have been documented among both adults^36^ and adolescents.^9^,^37^ Ross and Ivis^38^ found that adolescents who binged also used more alcohol, cannabis and other illicit drugs. Binge eaters reported getting drunk more often and drinking more at each sitting than adolescents who did not binge eat. Krahn and colleagues^39^ reported that the intensity of alcohol use and negative consequences associated with alcohol use were correlated with the severity of binging behaviors. Conason and Sher^9^ found that substance use was related to increased engagement in risky behaviors such as attempted suicide, delinquency and risky sexual behaviors among adolescents with bulimia nervosa, although not among adolescents with anorexia nervosa.

Substance use and ED symptoms may be linked by underlying factors such as impulsivity and/or emotional distress. Studies have shown association between alcohol use and bulimic behaviors in both men and women.^18^,^40^–^42^ Studies of adult women with comorbid EDs and substance use have been found to be more impulsive across multiple domains than adult women with BN only,^40^ although it is not clear that the same relationship exists among adolescents.^42^

Other studies suggest that adolescents with EDs and/or ED symptoms use alcohol to cope with problems caused by their EDs.^9^,^31^,^43^ Internalizing behaviors are frequently comorobid with disordered eating and EDs.^32^,^44^ Birch and colleagues^43^ report that heavy drinking or binge eating in isolation of each other often occur in situations that involve pleasant emotions, interpersonal interactions, and social pressure, whereas binge eating combined with heavy drinking is more likely to occur in situations that involve unpleasant emotions, physical discomfort or situations involving urges, temptations, and testing control.

Much of the existing literature on adolescents is based on epidemiological studies^23^,^26^,^27^,^29^ or studies of adolescent inpatients.^2^,^45^,^46^ This study utilizes a sample of adolescents in outpatient treatment for alcohol abuse or dependence. Previous studies describe the severity of substance use among adolescents categorized by whether or not they have an ED or subthreshold condition. 34,35,39 This study investigates the severity of ED symptoms and their association with drinking behaviors among a sample of adolescents with externalizing behaviors, who do not yet developed an ED.

We hypothesize that adolescents who report more ED symptoms will have greater severity of alcohol use as indicated by self-reported frequency of drinking to intoxication, socio-psychological and physiological problems associated with alcohol use. Consistent with the empirical evidence, we also hypothesize that the association to be greater among females than males and among adolescents who exhibit more symptoms associated with internalizing disorders such as depression. Chi Square tests and analysis of variance are used to examine the prevalence of ED symptoms in the sample with documentation of gender differences. Bivariate and multivariate analyses investigate the relationships between ED symptoms and problematic alcohol consumption, controlling for gender, age, and number of internalizing disorders.

## Method

### Sample

The study sample consisted of 177 consecutively admitted adolescents who participated in a randomized clinical trial to test the effects of aftercare following cognitive-behavioral therapy (CBT) for alcohol use disorders with or without other substance dependence at three treatment sites in Connecticut. Three subjects from the original study were excluded because data on psychiatric disorders was not available. Subjects were treatment-seeking adolescents recruited through advertisements and referrals from the community. Most (76.8%) of the sample was Caucasian (6.8% African American, 13.6% Latino/Hispanic, 2.8% other groups). One third of the sample was female. The average age was 16 years (sd = 1.20), with a range of 13–18 yrs. The majority were attending school (94%). Less than half (43.5%) had previous mental health treatment and 39% were taking prescription medications at entry to treatment. The sample predominately represents youths with middle class backgrounds who resided in suburban communities. Although limited data on socioeconomic status was collected, collateral data indicates that all of their mothers had graduated high school and a substantial number had some college education.

### Procedures

The study utilizes data collected during baseline assessments for a larger treatment study, which were obtained during face-to-face interviews conducted by a Master’s level research assistant prior to the beginning of the 9-session weekly CBT group program for AOSUDs. A 10-minute phone or in-person screening interview was used to determine study eligibility. To be included in the study, each youth had to meet all of the following criteria: a) 13–18 years of age at time of intake assessment; b) current DSM-IV^47^ diagnosis criteria for alcohol abuse or dependence; c) current potentially harmful drinking as evidenced by ethanol consumption (beer, wine, or distilled spirits) of 3 or more standard drinks per drinking day for males, and 2 or more standard drinks per drinking day for females, at least three times during the 90 days before treatment or meeting DSM-IV criteria for alcohol intoxication at least once during this period (unless child was recently in a controlled environment, at which point it should be 90 days prior to enrollment); d) willingness to accept treatment and random assignment to aftercare conditions; e) able to comprehend and read English at a fifth-grade level; f) residence within 45 minutes drive from the two treatment sites; and g) participant and a family member responsible for providing collateral information are not planning to move outside the range noted above during the next 6 months. Exclusion criteria included: a) meeting DSM-IV criteria for substance dependence other than alcohol, nicotine, or marijuana; b) meeting DSM-IV criteria for schizophrenia or other psychotic disorders; c) a current medical condition that prevented regular participation in treatment or aftercare; and d) exhibiting suicidal behaviors including suicidal ideations with a plan, serious self-injurious behaviors, a plan to harm others or harmful behavior to others in the past 30 days. Youths who met criteria for substance abuse were included.

### Measures

Data for the current analyses were obtained from the pre-treatment assessment battery which included the *Teen Addiction Severity Index* (T-ASI),^48^ Alcohol Consumption Questionnaire (ACQ),^49^ and the *Diagnostic Interview Schedule for Children (*DISC-IV).^50^

#### Sociodemographic characteristic

The T-ASI provided sociodemographic information. The T-ASI is a semistructured interview that was modified from the Addiction Severity Index^51^ to evaluate the severity of adolescent substance abuse and associated problem domains.

#### Psychiatric diagnoses

The DISC-IV was used to obtain current and life-time psychiatric diagnoses for eating and substance use disorders and symptom severity among youths as defined by the DSM-IV. All data on eating disorder symptoms, include weight and weight disturbance, were self-report. The number and type of ED symptoms (AN and BN) as defined by the DSM-IV were included in the analyses, regardless of whether the subjects met the full diagnostic criteria. The DISC-IV was also used to measure the number of symptoms associated with internalizing disorders such as depression.

#### Alcohol and drug use

Alcohol use was collected using the ACQ which asks respondents to self-report numbers of times they used alcohol to the point of intoxication in the last year, as well as social-psychological problems and physical symptoms resulting from alcohol consumption. Measures of alcohol consumption were recoded to the mean of the ordinal response categories and log transformed to adjust for skewness prior to multivariate analysis. Additional dependent measures included the sum of the number of social-psychological problems (potential range is 0 to 8 alcohol-related problems) and the sum of physical symptoms (potential range is 0 to 8 alcohol-related symptoms).

### Statistical analysis

The analyses were conducted in three steps. First, chi square tests were used to examine the prevalence of specific ED symptoms by gender since the literature documents gender differences in prevalence rates. Second, similar chi square tests were used to determine whether there were statistically significant differences in self-reported alcohol consumption measures based on the ACQ among adolescents who exhibited no ED symptoms versus those that exhibited 1 or more symptoms, excluding amenorrhea. Finally, ACQ responses on alcohol consumption during the year before treatment entry were transformed into continuous measures, times that the adolescent reported becoming intoxicated in the year prior to treatment entry, and alcohol-related social-psychological problems and physical symptoms. Separate multivariate linear regressions were conducted to test whether the association between ED symptoms and (1) alcohol consumption, (2) social-psychological problems resulting from alcohol use, and (3) alcohol-related physical symptoms remained robust after taking into account the effects of gender, age, and the number of internalizing symptoms. Multivariate analyses also tested for interaction effects.

## Results

### Prevalence rates

The prevalence of full syndrome EDs in the sample was low; no subjects obtained a lifetime or current diagnosis of AN. One youth (0.6%) qualified for a current diagnosis of BN and there were no other youths who exhibited current or lifetime diagnoses for BN. However, one fourth of the sample (n = 46 youths, 26.4%) exhibited ED symptoms (see Table 1). Among youths exhibiting ED symptoms, 19% (N = 33 youths) reported having only symptoms of bulimia nervosa, 6.9% (N = 12) reported having only anorexic symptoms, and 0.6% (N = 1) reported symptoms of both anorexia and bulimia nervosa. The average number of symptoms reported was 0.30 for anorexia nervosa (standard deviation (sd = 0.08) and 0.93 for bulimia nervosa (sd = 0.80). Thirty youths (17.2%) reported that their weight influenced their self-evaluations. Twelve youths reported being underweight and only one eating disorder symptom; one reported a refusal to maintain weight and 11 reported that weight influenced their self-evaluations.

### Gender differences in ED symptoms

Gender differences were observed. Although the only subject who met criteria for an ED was male, females were more likely to report ED symptoms than males (N = 28, 49.1% of females vs. N = 18, 15.3% of males, Chi Sq = 25.46, df = 1, p = 0.000). As Table 1 indicates, females were much more likely than males to report having symptoms of bulimia nervosa (N = 22 females, 38.6% vs. N = 11 males, 9.4%) and somewhat more likely to report having symptoms of anorexia nervosa (N = 5 females, 8.8% vs. N = 7 males, 6.0%) (Chi Sq = 25.64, df = 3, p = 0.000). However, there were no statistically significant differences in the average number of symptoms of anorexia nervosa or bulimia nervosa reported by gender. More females (N = 22, 38.6%) indicated that their self-evaluations were influenced by their weight compared to boys (N = 8, 6.8%) (Chi Sq = 27.09, df = 1, p = 0.000). Two girls (3.5%) and no boys reported refusal to maintain their body weight. Males and females were equally likely to report binge eating (N = 3, 2.6% of males and N = 3, 5.3% of females) and compensatory behaviors (N = 2, 1.7% of males and N = 3, 5.3% of females).

### ED symptoms and alcohol use at entry to treatment

Table 2 summarizes information on alcohol consumption from the ACQ. Adolescents with ED symptoms were more likely to report getting drunk weekly in the year before entering AOSUD treatment than those without ED symptoms (Chi sq = 6.24, df = 1, p = 0.012), although there were no statistically significant differences in the odds of drinking alcohol on a weekly or more basis or drinking heavily.

Almost all adolescents in the study reported alcohol-related social or psychological problems (N = 164, 94.2%). The most frequently reported problems associated with alcohol use were parents’ worry about the child’s alcohol use (64.8%), driving under the influence (64.1%) and problems with family members (53.1%). Adolescents with ED symptoms tended to report more often that their parents worried about their alcohol use (Chi sq = 6.42, df = 1, p = 0.011) and that they felt bad about their drinking behavior (Chi sq = 11.11, df = 1, p = 0.001). There was also a statistically significant difference in the average number of alcohol-related social-psychological problems reported by adolescents with ED symptoms (Mean = 4.17, sd = 1.69) compared to those without ED symptoms (Mean = 3.05, sd = 1.97) (F = 11.70, p = 0.001).

The majority of adolescents in the sample reported physical symptoms associated with excessive alcohol consumption during the year before entering treatment (N = 146, 83.9%), with an average of 2.64 (sd = 1.94) symptoms. There were no statistically significant differences in the number of symptoms reported between adolescents with and without ED symptoms. The same was true for most individual symptoms. The only observed difference was the likelihood of reporting having tremors or morning shakes (Chi sq = 4.68, df = 1, p = 0.030).

### Multivariate correlates

Table 3 summarizes the results of linear regression analyses to examine the relationship between ED symptoms on alcohol consumption measures at entry to treatment. The purpose of this analysis is to determine whether the association between ED symptoms and alcohol consumption remains after controlling for these covariates. Gender was included as covariates because the preceding analysis suggested different patterns of association might exist for males and females. Age was included as a covariate because the risk of problematic drinking patterns is likely to become greater with age. Finally, the number of symptoms of internalizing disorders was included to test for the possible effects of depression. Analyses tested for interaction effects between being female and depression and between the number of ED symptoms and the number of internalizing symptoms. Preliminary analyses indicated no statistically significant differences in number of ED symptoms or type across groups of subjects divided by race-ethnicity, and family characteristics. Correlation coefficients showed that ED symptom severity was associated with more internalizing symptoms (r = 0.40, p = 0.000), the frequency of getting drunk (r = 0.19, p = 0.011), socio-psychological problems (r = 0.24, p = 0.002) and physiological problems (r = 0.19, p = 0.000) in the last year. Multivariate findings (see Table 3) indicate that adolescents who reported more ED symptoms were more likely to report getting drunk weekly or more, had more social-psychological problems associated with their alcohol use, and reported more physical symptoms of alcohol misuse than adolescents without ED symptoms after controlling for the effects of age, gender, and internalizing symptoms. Age increased the severity of alcohol consumption patterns and consequences. Being female was related to more alcohol-related physical symptoms. The number of internalizing symptoms decreased moderately the frequency of getting drunk in the year before entering treatment. There was statistically significant interaction effect between being female and internalizing symptoms related to alcohol-related physical symptoms; girls in general reported more physical symptoms, but girls who reported more internalizing symptoms tended to report fewer alcohol-related physical symptoms than others in the sample. Analyses were run using only symptoms of BN since they were more prevalent than symptoms of AN. The results were very similar.

## Discussion

There are some limitations of the current study that warrant comment. The data analyzed derives from a randomized clinical trial that was designed to test the effectiveness of AOSUD treatment and aftercare for adolescents, not ED symptoms *per se*. EDs are low prevalence in the general population and were observed to be low in the study sample. Thus, the small sample size precluded testing of more complex multivariate models that may contribute to the understanding of ED symptoms among adolescents in treatment. However, in order to achieve statistical significance in this sample, effect sizes had to be large. Thus, the significant findings described are likely to be quite robust. The sample also was a convenience sample and may not be representative of other AOSUD adolescent populations, although the characteristics of the sample suggests that it is representative of adolescents in outpatient treatment for AOSUDs in Connecticut. Self-report measures of ED symptoms were collected as part of the diagnostic battery administered at baseline. Adolescents may have underreported ED symptoms due to stigma or embarrassment. Similarly, they may have inaccurately reported the frequency of behaviors. Despite these limitations, this study represents an important contribution to the literature by providing evidence on the relationship between ED symptoms in a clinical sample of adolescents who typically exhibit multiple externalizing symptoms. Better understanding of the underlying factors that contribute to both ED and AOSUD behaviors may help earlier identification of a subset of adolescents who need specific interventions.

The prevalence of EDs was very low and conclusions regarding EDs and substance use should be made with caution. We anticipated lower rates of EDs and related symptoms, since we recruited subjects for the study from local programs that offer a range of mental health and substance abuse treatments. It is likely that the recruitment sites would have made referrals to other services within their agency if EDs were suspected rather than refer to a study were they would receive primarily alcohol and drug treatment intervention. Individuals with co-occurring AOSUDs and full syndrome EDs would more likely be in an intensive outpatient, partial hospitalization, or inpatient program, rather than an outpatient research program, due to the severity of the combined illnesses.

Although the prevalence of full EDs was low in our sample, the fact that so many youths exhibited symptoms associated with EDs is clinically significant. Over one-fourth of the sample (15.3% of males and 49.1% of females) reported symptoms of EDs. This is especially relevant since adolescents who exhibit ED symptoms may be at an early stage in the development of the disorder and may later develop full EDs.^17^,^52^ The presence of ED symptoms can affect both the course of the development of AOSUDs,^9^,^12^–^14^ as well as their treatment.^15^

Our findings were consistent with other studies^53^ that demonstrate links between substance use and EDs, particularly BN and bingeing behaviors. We hypothesized that there would be a relationship between reporting more ED symptoms will have greater severity of alcohol use as indicated by self-reported frequency of drinking to intoxication, socio-psychological and physiological problems associated with alcohol use. Both bivariate and multivariate findings supported our hypothesis that youths with symptoms of eating disorders would exhibit more frequent alcohol use to intoxication and more problems associated with their alcohol use.

Findings partially supported the hypothesis that the relationship will be stronger for females than males. Rates of ED symptoms were higher among females than males. Females reported refusing to maintain their body weights and that their self-evaluations were affected by their weight more than males. However, the relationship between ED symptoms and some measures of alcohol consumption appeared to differ by gender. Multivariate findings found that gender was only related to alcohol-related physical symptoms; it was not associated frequency of getting drunk or alcohol-related social problems after taking into consideration the effects of ED symptoms, age, and internalizing symptoms. Additional descriptive analysis of gender and ED (not shown) suggested that there was a relatively small group of males (N = 18) who tended to get drunk more often and had more alcohol-related problems, compared to other males. These males with ED symptoms were more similar to females on many of the alcohol consumption measure. Females and males with ED symptoms tended to drink more heavily, get drunk more frequently, and feel worse about their drinking, than males without ED symptoms. Males with ED symptoms also reported getting arrested as a result of drinking alcohol with higher rates of DUI. Most strikingly, they were more likely to report have severe stomach problems and pass out as a result of alcohol use than other males. These rates were also higher than females. Although a definitive conclusion cannot be drawn based on the current analysis due to the small sample size, findings suggest that gender may play a moderating role in the relationship between increased ED symptoms, heavier drinking, and other drinking related symptoms. Further study is warranted.

Thus, our findings suggest that there is a subset of adolescents in AOSUD treatment that may be at risk of developing full EDs, especially BN. The ability to identify and address ED symptoms among AOSUD clients represents an opportunity to address ED-related behaviors within the context of AOSUD treatment and possibly circumvent the development of more severe ED-related problems.

Problematic alcohol consumption patterns and ED symptoms may also share some underlying causes, such as impulsivity and/or emotional distress. In this study, symptoms of BN were most common among both males and females who reported ED. Identifying partial ED syndromes may have clinical relevance, however, analysis of specific symptoms may reveal more about the relationship between EDs and AOSUDs. Identifying other factors that play a role in the expression of these symptoms, such as levels of impulsivity, or specific personality factors, may help better explain the relationship between eating and substance use disorders. In adult studies, females with BN and alcohol dependence are more impulsive across a variety or domains than females with BN alone.^40^ Wonderlich et al^42^ found a link between behavioral measures of impulsivity (specifically substance use and delinquency) and the onset of purging behavior, although there was no connection on measures of trait impulsivity in the sample of adolescent females. Despite the initial negative finding in adolescents by Wonderlich et al,^42^ trait measures of impulsivity such as reward and punishment sensitivities, and putative endophenotypic measures such as P300 electrophysiologic response or impaired set-shifting may be helpful in deconstructing the link between EDs and AOSUDs and should be investigated further.^54^–^56^

Prospective studies of adolescents may be able to examine the impact of EDs on the course of AOSUDs and vice-versa. Further defining the trajectory of illness for EDs and their symptoms, with and without AOSUDs, is critical. More efforts should be directed at identifying youths with co-morbid ED/AOSUD pathology in treatment programs, who may need more intensive interventions. More research exploring the elusive but clinically important relationship between substance use and individual eating disorder symptoms is warranted. Further exploration of the relationships and pathogenesis between these co-occurring disorders may enrich our understanding of the pathogenesis of psychiatric illness, and could potentially improve assessment, and treatment of these disorders.

## Figures and Tables

**Table 1:** Self-reported eating disorder symptoms by gender.^1^

		Total (N = 174)	Males (N = 117)	Females (N = 57)	Significance
***Specific symptoms***					
Underweight	N	15	9	6	*χ*^2^ = 0.39, df = 1, p = NS
	%	8.6	7.7	10.5	
Refusal to maintain body weight	N	2	0	2	*χ*^2^ = 4.15, df = 1, p = 0.042
	%	1.1	0.0	3.5	
Intense fear of becoming fat	N	0	0	0	
	%	0.0	0.0	0.0	
Body weight disturbance	N	12	7	5	*χ*^2^ = 0.42, df = 1, p = NS
	%	7.0	6.1	8.8	
Amenorrhea	N	N/A	N/A	28	
	%	N/A	N/A	49.1	
Recurrent episodes of binge eating	N	6	3	3	*χ*^2^ = 0.84, df = 1, p = NS
	%	3.4	2.6	5.3	
Compensatory behavior	N	5	2	3	*χ*^2^ = 1.73, df = 1, p = NS
	%	2.9	1.7	5.3	
Binging and purging 2 times per week for 3 months	N	2	1	1	*χ*^2^ = 0.27, df = 1, p = NS
	%	1.1	0.9	1.8	
Self-evaluation influenced by weight	N	30	8	22	*χ*^2^ = 27.09, df = 1, p = 0.000
	%	17.2	6.8	38.6	
***Total with any ED symptoms***	N	46	18	28	*χ*^2^ = 25.46, df = 1, p = 0.000
	%	26.4	15.3	49.1	
AN symptoms only	N	12	7	5	*χ*^2^ = 2.97, df = 2, p = NS
	%	26.1	38.9	17.9	
BN symptoms only	N	33	11	22	
	%	71.7	61.1	78.6	
AN and BN symptoms	N	1	0	1	
	%	2.2	0.0	3.6	

1 Listwise deletion of missing data was used.

**Table 2:** Alcohol consumption and related problems by ED symptoms.^1^

		Total (N = 174)	No ED Sx (N = 128)	ED Sx (N = 46)	Significance
***Alcohol consumption***					
Drank alcohol weekly or more	N	94	65	29	*χ*^2^ = 2.05, df = 1, p = NS
	%	54.0	50.8	63	
Drank heavily weekly or more	N	79	54	25	*χ*^2^ = 2.05, df = 1, p = NS
	%	45.4	42.2	54.3	
Got drunk weekly or more	N	54	33	21	*χ*^2^ = 6.24, df = 1, p = 0.012
	%	31.0	25.8	45.7	
***Social-psychological problems***					
Problems with parents/family	N	100	68	32	*χ*^2^ = 3.74, df = 1, p = 0.053
	%	57.5	53.1	69.6	
Parents worried about alcohol use	N	122	83	39	*χ*^2^ = 6.42, df = 1, p = 0.011
	%	70.1	64.8	84.8	
Felt bad about drinking	N	92	58	34	*χ*^2^ = 11.11, df = 1, p = NS
	%	52.9	45.3	73.9	
Trouble at school/work	N	38	29	9	*χ*^2^ = 0.19, df = 1, p = 0.001
	%	21.8	22.7	19.6	
Neglected obligations	N	56	32	24	*χ*^2^ = 11.45, df = 1, p = 0.001
	%	32.2	25	52.2	
Sought help	N	35	22	13	*χ*^2^ = 2.58, df = 1, p = NS
	%	20.1	17.2	28.3	
Arrested because of drinking	N	26	17	9	*χ*^2^ = 1.05, df = 1, p = NS
	%	14.9	13.3	19.6	
Driving under the influence	N	114	82	32	*χ*^2^ = 0.45, df = 1, p = NS
	%	65.5	64.1	69.6	
***Physiological problems***					
Severe stomach problems	N	39	22	17	*χ*^2^ = 7.61, df = 1, p = 0.066
	%	22.4	17.2	37	
Vomited blood	N	6	4	2	*χ*^2^ = 0.15, df = 1, p = NS
	%	3.4	3.1	4.3	
Nausea/severe hangover	N	88	61	27	*χ*^2^ = 1.65, df = 1, p = NS
	%	50.6	47.7	58.7	
Tremors/morning shakes	N	22	12	10	*χ*^2^ = 4.68, df = 1, p = 0.030
	%	12.6	9.4	21.7	
Alcohol-induced headaches	N	117	87	30	*χ*^2^ = 0.12, df = 1, p = NS
	%	67.2	68	65.2	
Blackouts	N	68	50	18	*χ*^2^ = 0.00, df = 1, p = NS
	%	39.1	39.1	39.1	
Difficulty concentrating	N	38	25	13	*χ*^2^ = 1.51, df = 1, p = NS
	%	21.8	19.5	28.3	
Passing out	N	82	57	25	*χ*^2^ = 1.30, df = 1, p = NS
	%	47.1	44.5	54.3	

* *p* ≤ 0.05.

** *p* ≤ 0.001.

1 Listwise deletion of missing data was used.

**Table 3:** The relationship of the number of eating disorder symptoms and drinking behaviors (N = 174).^1^

	Times got drunk in last year			Alcohol-related social problems			Alcohol-related physical symptoms		
			
	b	SE	β	b	SE	β	b	SE	β
Age	0.20*	0.08	0.18	0.27*	0.12	0.16	0.24*	0.12	0.15
Female	0.22	0.24	0.08	0.59	0.32	0.14	1.88**	0.58	0.46
Number of ED symptoms	0.52*	0.17	0.25	0.60*	0.24	0.19	0.62*	0.25	0.20
Number of internalizing symptoms	−0.02*	0.01	−0.19	–	–	–	0.02	0.01	0.21
Interaction (Female*Internalizing Sx)	–	–	–	–	–	–	−0.06**	0.02	−0.55
N	173			173			173		
Intercept	−0.17			−1.33			−0.97		
R square	0.10*			0.10**			0.06*		
Standard error of the estimate	1.30			1.88			1.90		

* *p* ≤ 0.05.

** *p* ≤ 0.001.
